# Fluorescence Microscopy of Superplasticizers in Cementitious Systems: Applications and Challenges

**DOI:** 10.3390/ma13173733

**Published:** 2020-08-24

**Authors:** Johannes Arend, Alexander Wetzel, Bernhard Middendorf

**Affiliations:** Department of Structural Materials and Construction Chemistry, University of Kassel, Mönchebergstr. 7, 34125 Kassel, Germany; alexander.wetzel@uni-kassel.de (A.W.); middendorf@uni-kassel.de (B.M.)

**Keywords:** superplasticizer, polycarboxylate ether (PCE), fluorescence microscopy, retardation, C_3_S, calorimetry, adsorption, slump

## Abstract

In addition to the desired plasticizing effect, superplasticizers used in high and ultra-high performance concretes (UHPC) influence the chemical system of the pastes and for example retardation of the cement hydration occurs. Thus, superplasticizers have to be chosen wisely for every material composition and application. To investigate the essential adsorption of these polymers to particle surfaces in-situ to overcome several practical challenges of superplasticizer research, fluorescence microscopy is useful. In order to make the superplasticizer polymers visible for this microscopic approach, they are stained with fluorescence dyes prior the experiment. In this work, the application of this method in terms of retardation and rheological properties of sample systems is presented. The hydration of tricalcium oxy silicate (C_3_S) in combination with different polycarboxylate ether superplasticizers is observed by fluorescence microscopy and calorimetry. Both methods can identify the retarding effect, depending on the superplasticizer’s chemical composition. On the other hand, the influence of the superplasticizers on the slump of a ground granulated blast furnace slag/cement paste is correlated to fluorescence microscopic adsorption results. The prediction of the efficiency by microscopic adsorption analysis succeeds roughly. At last, the possibility of high-resolution imaging via confocal laser scanning microscopy is presented, which enables the detection of early hydrates and their interaction with the superplasticizers.

## 1. Introduction

Modern high and ultra-high performance concretes base upon a dense matrix resulting in superior mechanical properties such as compressive strength and durability [1,2]. Fine fillers such as silica fume and a very low water content are essential to achieve this dense matrix and thus properties. The combination of a high amount of fines and little amount of mixing water causes some challenges concerning mixing and application of those concretes because particles agglomerate and no homogeneous paste can be obtained without additives [3]. So-called plasticizers or water-reducing agents (superplasticizers, SP) achieve the issue of attractive forces between the particles, inducing a well dispersed suspension even in less water [4]. Today, the most popular and effective superplasticizers are based on polycarboxylate ethers (PCE), invented by Hirata [5]. With a side chain carrying, negatively charged carboxylate-backbone, the polymers adsorb to mineral surfaces and shield electrostatic forces, while the polyether-sidechains induce a steric hindrance to separate the fine particles. The adsorption of the polymers is very important for the plasticizing effect and is influenced by various parameters, especially by the aqueous phase in terms of present ions and their concentration and the mineral composition of the solid particles. Those parameters determine the zeta-potential of the particles, thus the adsorption of the polymers, and, in turn, considering the chemical structure of the PCE, the effectiveness of the superplasticizer for the certain system. While the plasticizing effect is highly desired, the presence of most SP-polymers retards the hydration of reactive phases [6,7,8,9]. One discussed reason is the formation of polymer layers, which block mineral surfaces (e.g., kinks) and interfere on the one hand the dissolution of reactive species and on the other hand the precipitation of hydration products [10,11,12,13,14,15,16]. The investigation of the superplasticizer adsorption is thus twice important to estimate or predict the overall performance for certain applications. Since these superplasticizers were invented, at lot of research has been carried out to correlate the characteristics of the PCEs in different cementitious systems to their chemical structure. However, in the last decades, findings were either quite superficial or rather specific to the observed system [4,17,18,19,20,21,22,23,24,25,26]. To make new experimental data accessible and reduce the effort of empirical studies, the authors invented a microscopic in-situ approach for SP-investigation [27,28]. The fluorescence microscopy allows insights with respect to different aspects: On the one hand, the fluorescence intensity of particles in suspensions with stained superplasticizers correlates with the amount of adsorbed polymer and is an indicator for the plasticizing performance of the investigated material combination. On the other hand, dissolution and precipitation of solids can be visualized by time depending changes of fluorescence images, which usually fails with ordinary light microscopy because of bad contrasts or transparency of phases. Experiments and results for both aspects, plasticizing and retardation, are presented in the following with focus on the layer formation of adsorbed polymers. The method enables the localization and quantification of adsorbing polymers in-situ and within very little samples, which makes it a useful addition to common approaches of adsorption measurements such as the depletion method using the determination of the residual carbon in solution (TOC). Further, this method can be extended to investigate mixtures of different SPs within on sample and visualize for example competing adsorption, which is a unique feature of this approach. Specific interactions with different phases are also in the scope for advanced investigations. In this context, the present work lays the foundation for extended research.

Ordinary Portland cement (OPC) itself is composed of different clinker phases, setting agents and grinding additives, thus even a plain mixture of cement and water is a quite complex system to investigate superplasticizers. Considering approaches to reduce the usage of OPC applying supplementary cementitious materials (SCM) to produce more climate friendly concretes, the variation of compositions further increases and assumptions of the suitability of (also) very diverse chemical composed superplasticizers usually requires empiric studies. In order to reduce time and material consuming experiments, prediction approaches need to be found. Interesting results were achieved by Marchon et al. [29] showing the relation of the molecular structure of SPs and their plasticizing and retarding effect to a model clinker. In the next step, the best chemical structure in terms of best plasticizing and least retardation was predicted successfully by analyzing experimental data for a defined clinker system [9]. A similar approach was reported by Cook et al. [30] and computerized simulations are getting more and more attention [31,32,33]. Lange et al. investigated the correlation of the hydrophilic–lipophilic balance (HLB-value) SP-induced rheological properties of cement pastes and concretes [34,35]. Further examples could be mentioned concerning the optimization of superplasticizers for different applications, but nevertheless general predictions will probably fail facing the complex material systems and possible polymer structures. In this context, the fluorescence microscopic approach is a useful tool to reduce the effort for superplasticizer investigation and thus optimization, because the possibility to localize and quantify superplasticizers in-situ enables a new way to setup performance prediction models.

Previous research indicated a strong influence of the used SP to the dissolution speed of gypsum grains in cementitious model systems [28]. The results correlate with calorimetric measurements of the retarded hydration in presence of those SPs and provided arguments for the concept of a retarding polymer layer on reactive surfaces, because the retardation decreased, when silica fume was added and thus the specific surface was enlarged [36]. In this work, tricalcium oxy silicate (C_3_S, alite) as pure clinker phase is used to get specific results concerning the formation of calcium silicate hydrate phases (C-S-H) by the hydration of the clinker in dependency of SP-type and concentration, to estimate different approaches of the SP’s retardation potential. Correlations to calorimetric and electron microscopic studies support the findings of the fluorescence microscopic experiments.

In terms of rheological properties, the influence of different PCEs to pure und blended cement pastes was taken into account. The adsorption of SPs to cement and ground granulated blast-furnace slag (GGBFS) was detected by fluorescence microscopy and compared to the results of slump experiments of the corresponding pastes. If the expected relation of high adsorption and good rheology of those systems could be supported by fluorescence microscopic results had to be proven.

Early hydration products are known to have an important impact on the superplasticizer performance because they use to adsorb high amounts of SP-polymer due to positive surface charges and extension of the specific surface area [37] or even incorporate the molecules in their crystal structure [38]. The presence of specific minerals such as clay [39,40] or an unfortunate combination of SP and binder materials [41] may lead to a complete loss of the plasticizing effect of SPs. In this context, high-resolution fluorescence microscopy via confocal laser scanning microscopy [42,43] allow a precise detection of those phases and the adsorption of SPs on cement grains, as shown below. In combination with an extensive mineralogical analysis of the substrates, this method enables interesting in-situ experiments to investigate the very early hydration process and SP influence. First images of such experiments are finally presented.

## 2. Materials and Methods

### 2.1. Solids

For the first part of this work, pure tricalcium oxy silicate is investigated. It was synthesized at the department of structural materials at the Bauhaus-University of Weimar (Germany) by melting stoichiometric amounts of calcium carbonate (CaCO_3_) and silicon oxide (SiO_2_) at 1600 °C [44]. The final content of calcium oxide (CaO) was below 0.2 wt%, achieved by repeated grinding and heating. The specific surface area of the material was determined via nitrogen adsorption (BET-method) to 0.6 m^2^/g. The X-ray diffractogram is given in the Supplementary Materials (Figure S1).

The hydration of tricalcium oxy silicate with water (H respectively H_2_O) to C-S-H phases and portlandite (CH, Ca(OH)_2_) generally follows the equation [45]:

(1) $$C_{3}S+\left(3-x+z\right)\;H\;\to \;C_{x}SH_{z}+\left(3-x\right)\;CH$$

In Equation (1), x represents the C/S-ratio, usually varying between 1.2 and 2.1. n is the H/S-ratio in the formed C–S–H-phase.

To increase the specific surface area within the samples for calorimetric experiments, silica fume silicoll P (Sika, Baar ZG, Switzerland) with a specific surface area (BET) of ~20 m^2^/g was used. The SiO_2_-content is ~96% (see X-ray fluorescence analysis (XRF), Table 1).

The rheological experiments were conducted with an OPC, Type CEM I 52,5 R-SR 3 (na) (Holcim, Hamburg, Germany). The phase composition analyzed via X-ray diffraction (XRD) is given in Table 2. For XRF of oxides, see Table 1. The specific surface area (BET) is 1.1 m^2^/g.

As SCM, a GGBFS (Holcim, Salzgitter, Germany) with a specific surface area (BET) of 0.8 m^2^/g was used. The slag is a byproduct of the iron extraction process, mainly composed of CaO, SiO_2_ and Al_2_O_3_. The material is mostly amorphous if sufficiently cooled after production. Finely ground, a latent hydraulic binder is obtained. The composition of oxides is shown in Table 1.

### 2.2. Superplasticizers

**Figure 1:**
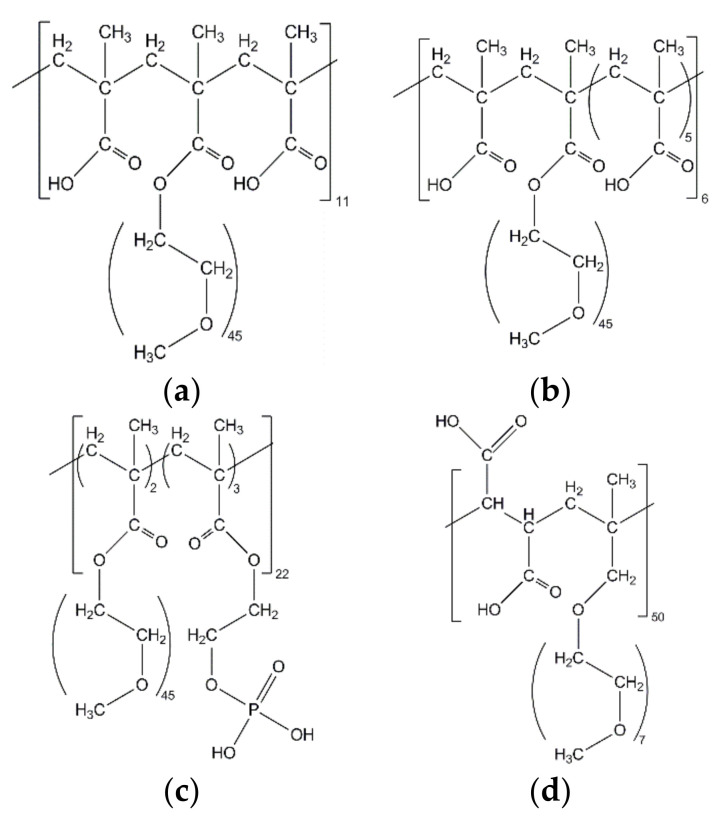
Chemical structure of the used SPs: (**a**) PC2; (**b**) PC6; (**c**) Phos3; and (**d**) APEG.

The superplasticizers were synthesized and characterized by the research group of Prof. Dr. Johann Plank at the Department of Chemistry at Technical University Munich, Germany. Four different SPs were used: two so-called MPEG-PCEs (PC2 and PC6) [46], a phosphor-PCE (Phos3) [47] and an APEG-type [48]. The MPEGs are copolymers of meth acrylic acid (charge carrying groups) and methoxy polyethylene glycol units (sidechain carrying groups). The length of the sidechains is 45 ether units in the case of PC2, PC6 and Phos3. PC2 (Figure 1a) has a monomer ratio of two methacrylic acids to one methoxy polyethylene glycol, yielding a PCE with a relatively high side chain and low charge density. PC6 (Figure 1b) is composed of six meth acrylic acids per methoxy polyethylene glycol group, thus there are few sidechains and a higher charge density [25]. The third PCE contains phosphate groups as charge carriers (Phos3, Figure 1c), polymerized with methoxy polyethylene glycol units in a ratio of 3:1. According to Stecher et al. [47], the real ratio is lower because the phosphate monomer is less reactive and does not react completely during the polymerization. The APEG superplasticizer (Figure 1d) is composed of maleic anhydride monomers and allylether-based sidechain carrying groups [49]. These monomers do not homopolymerize so the polymer has a monomer ratio of 1 [50]. The sidechain length is seven ether units, thus rather short. The two carboxyl groups in the maleic anhydride are responsible for a high charge density.

All superplasticizers were stained with the fluorescence dye amino-fluorescein (Merck, Darmstadt, Germany) following the same procedure, described in detail by Arend et al. [28]. The bond between polymer and dye was proven by fluorescence spectroscopy [28]. For calorimetric studies, the original SP-solutions were used with polymer contents given in Table 3.

### 2.3. Fluorescence Microscopy

Although the used fluorescence stereoscope M 205 FA (Leica Microsystems GmbH, Wetzlar, Germany) has limited resolution, diluted suspensions can be investigated reliably. Following the descriptions of Arend et al. [27,28], small amounts of mineral particles (1–2 mg) with various composition of the aqueous phase are sufficient for adsorption experiments. To enhance the contrast of the images, only coarse particles of the investigated solids are used. Therefore, the powders were washed with isopropanol and decanted five times. The mineral composition was verified via XRD and no deviation to the original materials was found. To obtain images of thin particle layers, the water/binder (*w*/*b*) ratio was set to 5. The concentration of the superplasticizers in the measuring solution was calculated to obtain 0.2%, 0.5% and 1.2% by weight of binder (%bwob). For example, 50 µL of a SP-solution with a concentration of 1 mg/mL were added to 10 mg substrate to achieve a ratio of 0.5 %bwob. The fact that the ratios of dissolved superplasticizer to binder are kept constant over all compared experiments but the ratio of SP-polymer to specific mineral surface area deviates from pastes of the raw material needs to be kept in mind and is discussed below. Synthetic pore solution [51] (composition is given in Supplementary Materials, Table S1) was used as liquid phase to allow clinker hydration even in this diluted system. The suspensions were intensively mixed by shaking in an Eppendorf tube. Three microliters of these suspensions were placed between the microscopic slides of the sample holder. As fast as possible (approximately 1 min after mixing), the investigation was started. The hydration of C_3_S was analyzed for 48 h and every 30 min a fluorescence image was taken automatically. The adsorption experiments were carried out by taking images right after sample preparation. Within the time scale, that is relevant for mixing real pastes (<1 h), no significant changes were observed. To quantify the SP-adsorption via fluorescence, the images were analyzed with imageJ (version 1.52n). After the subtraction of the background, the mean value of the residual signal from the particles was determined.

To enhance the resolution of the fluorescence microscopy, proof-of-principle-experiments were conducted with the confocal laser scanning microscope (CLSM) LSM880 Airyscan (Zeiss, Jena, Germany), using an argon laser with an excitations wavelength of 488 nm. Therefore, suspensions of raw OPC were prepared in the same way as for the other microscopic experiments. Comparable to a scanning electron microscope, this device images the sample by point-by-point laser excitation and measurement of the emitted fluorescence signal. Confocal microscopes are able to image three-dimensionally as far as the sample is transparent for excitation and fluorescence radiation by stacking 2D-scans of different heights. The better resolution allowed imaging without separation of the fine powder fraction, which might be a very important issue, as discussed below. Owing to the setup of the CLSM filigree microscopic slides had to be used, thus missing sealing and limited measure time forbid long-time investigations.

### 2.4. Scanning Electron Microscopy

The scanning electron microscopy (SEM) Quanta FEG 250 (FEI, New York, NY, USA) was used to visualize the hydration progress of C_3_S. After 48 h of fluorescence microscopic observation, the C_3_S-samples were investigated via SEM to identify C-S-H phases and prove the relation between portlandite precipitation and hydration. Therefore, the observed suspensions were dried at 40 °C and used without further preparation on a microscopic slide. The secondary electron mode was used with an acceleration voltage of 15 and 5 kV. The chamber pressure was 0.5 mbar.

### 2.5. Calorimetry

The most common way to investigate the hydration of reactive inorganic systems is to measure the heat flow that is produced by the physicochemical reaction. To get an overview of different experimental parameters, similar samples were prepared and the influence of SP-type, SP-concentration, composition of aqueous phase (tap water or synthetic pore solution (syn. PS)) and presence of silica fume were investigated as well as samples without PCE addition as reference. When using superplasticizers, the w/b ratio was kept at 0.25 and the water content of the superplasticizer solution was considered in the total amount of mixing water. To enable a homogenous mixture in absence of SPs, the *w*/*b* ratio was 0.4. The detailed sample composition is shown in Table 3. The amount of SP in given in the mass of obtained SP-solution. The isothermal calorimeter MC-CAL/100P (C3, Haar, Germany) was used for the heat flow measurements at the temperature of 20 °C.

### 2.6. Slump Test

The measurement of the slump according to standard DIN EN 1015-3 was conducted to estimate the rheology of a pure cement (OPC) paste and a 50:50 mixture of cement and GGBFS depending on the used superplasticizer type. A conus with a bottom diameter of 10 cm is placed in the middle of a horizontal even metal plate and filled with 300 mL of freshly mixed paste. The slump of the paste on the plate can be measured after the conus has been removed and spreading stopped. The diameter of the completely spread mortar is used to quantify the flowability. To compare the plasticizing effect of the four superplasticizers and the influence of SCM, pure and blended cement pastes were mixed. The w/b ratio was set to 0.25 and, although no silica fume was used, the high SP-dosage of 1.2% was chosen, to obtain results even with the less effective SP. The composition of the pastes is given in Table 4. As for the calorimetric samples, the water content of the SP solution was included to the mixing water calculation.

## 3. Retardation of C_3_S-Hydration–Results and Discussion

The influence of different SPs on OPC was investigated by Arend et al. [27]. In the same manner, the influence of SPs on pure tricalcium oxy silicate (C_3_S) was investigated here. This way, the hydration following Equation (1) without phenomena caused by other components of OPC could be investigated. In this study, the SP-type as well as the concentration was varied. To reduce the required testing time, SP-type with constant ratio of 0.5%bwob and different ratios of PC2, which caused the least retardation, were compared.

### 3.1. Fluorescence Microscopic Investigations

**Figure 2:**
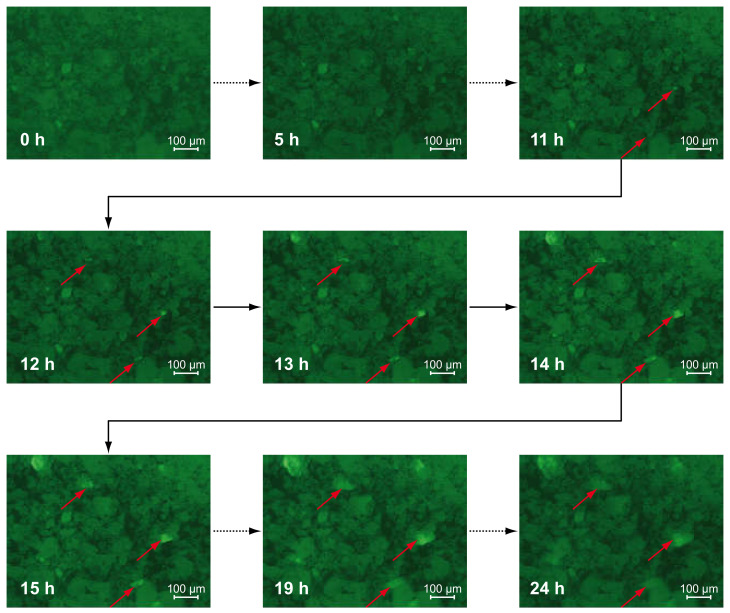
Fluorescence images of C_3_S-particles with 0.5% PC2 in synthetic pore solution over time with precipitating portlandite (arrows).

Representing the progress of the hydration, the formation of portlandite crystals can be measured in time dependence [52]. Because portlandite shows high SP-affinity resulting in strong signals, the progress of hydration can be measured by fluorescence microscopy, too. C-S-H phases are too small to be resolved in this experimental setup but will be shown by SEM imaging. Using synthetic pore solution, the hydration process is accelerated generally, because the saturation and precipitation concentration of hydrate-phases-forming, dissolving ions is reached earlier due to the already high concentration of ions (see also Section 3.3). For the fluorescence microscopic investigations, the time of the first image that shows portlandite precipitation is determined as the experiment’s outcome. When portlandite precipitates, the hydration is ongoing and C-S-H forms parallel Equation (1). In Figure 2, the series of fluorescence images of C_3_S with 0.5% PC2 in synthetic pore solution is shown, representatively. During the first hours (0–5 h), the contrast of the images increases slightly due to an adsorption of SP, in the beginning dissolved (background signal), to the particles. In this case, after a period of 11 h, first bright signals appear in the images, growing further to characteristic hexagonal crystals.

### 3.2. Scanning Electron Microscopic Investigations

**Figure 3:**
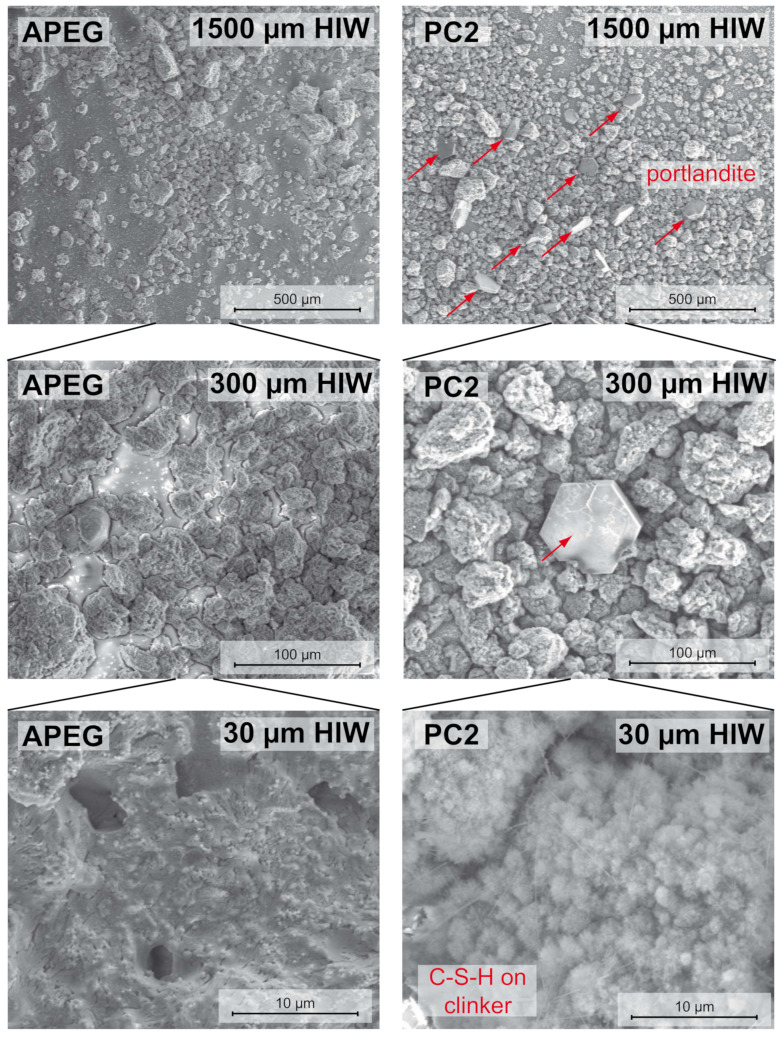
SEM-images of dried APEG- and PC2-samples (48 h after mixing) with: 1500 µm (**top**); 300 µm (**middle**); and 30 µm (**bottom**) horizontal image width (HIW).

The high resolution of the SEM was used to compare the surfaces of C_3_S-particles dispersed in different SP-solutions for 48 h. In the case of APEG, neither precipitation of portlandite via fluorescence microscopy nor any heat via calorimetry could be detected. Figure 3 shows the comparison of SEM images of APEG and PC2 samples at different magnifications. In the overview images (1500 and 300 µm horizontal image width (HIW)), the portlandite crystals are clearly recognizable. They are absent completely in case of APEG.

The high resolved images of the particle surfaces (30 µm HIW) indicate the expected difference: On the sample with APEG, no cementitious reaction products can be observed. The present precipitates might be residuals from dried pore solution. In contrast, in the case of PC2 a characteristic carpet of crystal needles appears–C-S-H-phases.

### 3.3. Calorimetry

**Figure 4:**
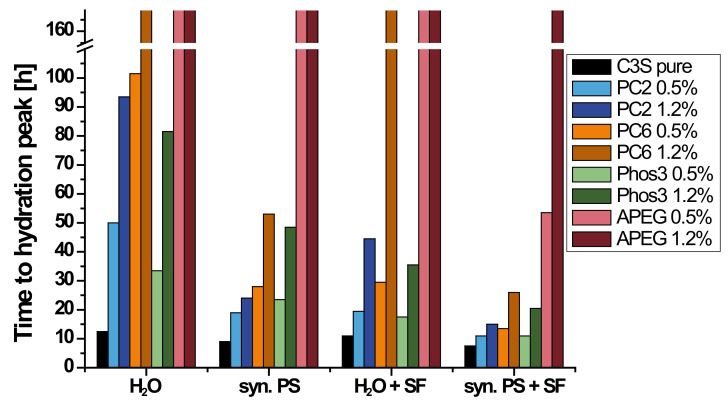
Illustration of hydration peaks depending on liquid phase (water (H_2_O) or synthetic pore solution (syn. PS)) and with or without silica fume (SF).

The results, meaning the time to reach the maximal hydration heat, of the calorimetric investigation (seven days) of various C_3_S/SP-samples are given in Figure 4 (data table can be found in Supplementary Materials, Table S2). Type and concentration of SPs were varied as well as the composition of the liquid phase. Additionally, the impact of increased SP-adsorbing surface area was investigated by adding silica fume (SF). For several samples, no heat development could be measured. Thus, no hydration occurred under these conditions. The time of maximal hydration heat was set to >168 h (seven days), but these samples were plastic even after several weeks in a closed vial, so the hydration seems to be inhibited at least for an unusually long period of time (especially in presence of APEG).

**Figure 5:**
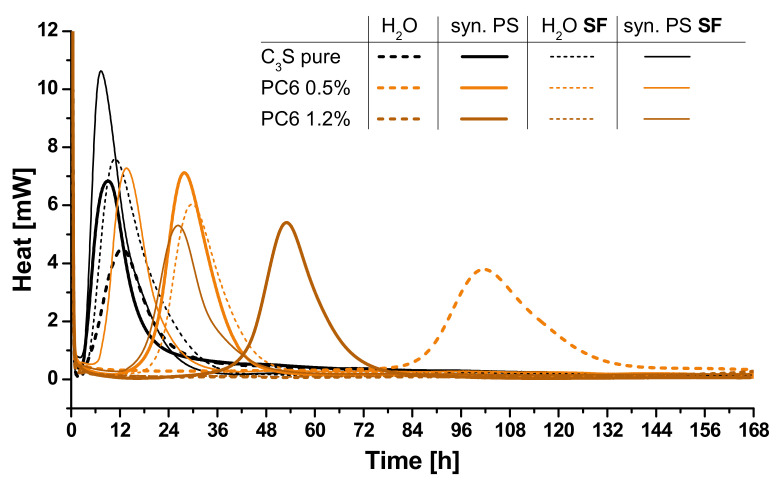
Heat flow of C_3_S-samples in water (dashed lines), synthetic pore solution (syn. PS) and with and without silica fume (SF, thick and thin lines) without SP addition (black), with 0.5% bwob (orange) and with 1.2% bwob (brown) PC6.

The development of the hydration heat is shown for pure C_3_S and samples with PC6, representatively, in Figure 5. The accelerating effect of synthetic pore solution and silica fume is indicated by strong peak shifts to earlier times. On the other hand, 1.2% bwob of PC6 in water inhibits the hydration extremely and no heat development is measured during the measuring period (thick brown dashed line). Without increased ion concentration and thus relieved hydrate precipitation (syn. PS) or increased specific surface area of the system (silica fume), the polymer layer inhibits any reaction between water and clinker in the first week after mixing.

It can be seen that the presence of synthetic pore solution affects the retardation of the SPs differently. While the retardation of PC2 and PC6 decreases strongly in comparison to dispersions in water, Phos3 shows a reduction in pore solution as well, but less pronounced. This is probably due to the different adsorption characteristics of the phosphate groups. The presence of silica fume reduces the retardation of all SPs in similar ways, suggesting a comparable relation between specific surface area and polymer coverage.

The extreme case of retardation is shown by APEG. Only if silica fume increases the specific surface area and synthetic pore solution is used in combination with a SP-dosage of 0.5%, the C_3_S hydrates after about two days (Figure 4). As described by Arend et al. [28], APEG is able to cover high amounts of specific surface area, still forming impermeable layers and water is hindered to reach the surface for several weeks. Properly, the short side chains allow a dense layer additionally to the high charge density of APEG [49]. This finding is an argument for the plausibility of layer-caused retardation. The strong interaction of silica fume with PCE-molecules, especially in presence of calcium ions [53,54], reduces the residual SP amount, which is able to passivate the reactive phases.

### 3.4. Correlation of Fluorescence Microscopy and Calorimetry

**Figure 6:**
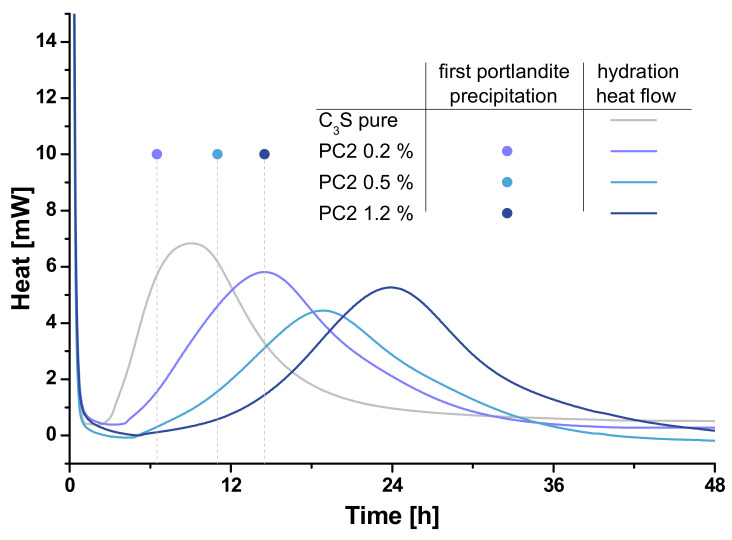
Fluorescence microscopic determination of first portlandite precipitation (dots) and heat flow (lines) of C_3_S-samples without (grey) and with 0.2% bwob (light blue), 0.5% bwob (blue) and 1.2% bwob (dark blue) of PC2.

**Figure 7:**
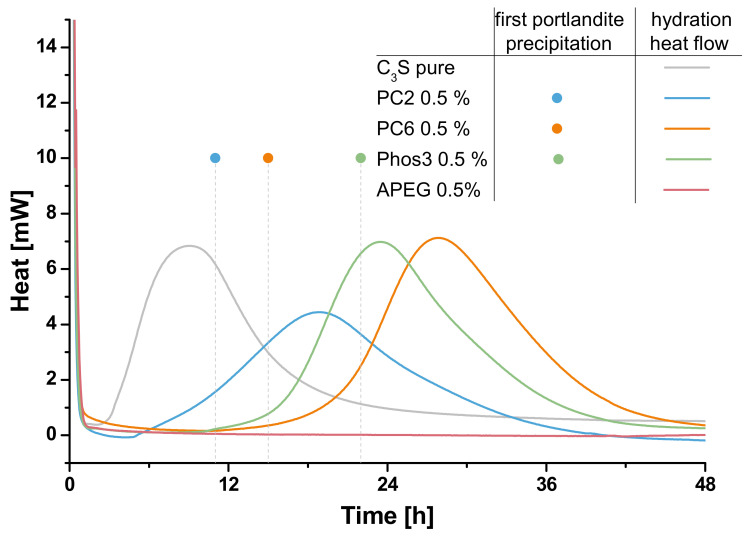
Fluorescence microscopic determination of first portlandite precipitation (dots) and heat flow (lines) of C_3_S-samples without (grey) and with 0.5% bwob PC2 (blue), PC6 (orange), Phos3 (green) and APEG (red).

The conducted fluorescence microscopic experiments as well as the calorimetric data allow an evaluation of the hydration of the clinker. Figure 6 and Figure 7 compare the results of both methods.

In general, the correlation of calorimetry and the estimation of first portlandite appearance in the fluorescence microscopy fits quite well because the relation of the retardation within every approach is well comparable. The increasing retardation caused by increased SP-concentration is remarkable in both data. For sure, the absolute times cannot be compared directly because on the one hand the precipitation of portlandite occurs earlier than the maximal heat flow is reached and on the other hand the above-mentioned difference in the specific surface area to SP-content ratio influences this parameter. Nevertheless, the fluorescence microscopic approach reflects the retardation reliably. Comparable results are reported using prevalent methods such as in-situ XRD [7] and nuclear magnetic resonance spectroscopy (NMR) [55]. The benefits of the fluorescence microscopic approach are the flexible experimental setup which allows, for example, the manipulation of the liquid phase during one experiment or the parallel detection of multiple polymer types, if stained with different dyes.

In the case of varied SP-types, the correlation does not work as well as in the case of concentration variation. Although PC6 shows a higher retardation in the calorimetric measurement than Phos3, the first precipitation of portlandite is detected much later in the case of Phos3. The reason for this might be the different chemical composition of the SPs, namely the potential of coordinating ions [47]. Because the ratio of synthetic pore solution and thus calcium ions to mass of SP is much higher in the fluorescence microscopic experiments, it is possible that the phosphate groups of Phos3 retard the precipitation of portlandite because calcium ions are coordinated to a higher degree than in the case of PC6. In the calorimetric experiments, this issue is less important because the content of synthetic pore solution is much less while the amount of SP, due to its relation to the mass of the clinker, is constant. In this case, all discussed reasons for the retardation of hydration by SP-polymers have to be considered though [56]:

- The passivation of reactive clinker, influenced by charge density and side chain length
- The shift of precipitation equilibria by the different ion coordination capacity
- The influence on the hydrate precipitation, as well determined by the chemical composition

More detailed investigations concerning time and optical resolution, for example via CLSM, may reveal further understanding of the interaction of those three retardation mechanisms.

## 4. Rheology of Pastes—Results and Discussion

To predict the efficiency of SPs for certain materials by fluorescence microscopy in the future, the amount of adsorbed SP determined by analysis of fluorescence images are correlated to the slump of corresponding pastes of cement.

### 4.1. Fluorescence Microscopic Adsorption Measurements

**Figure 8:**
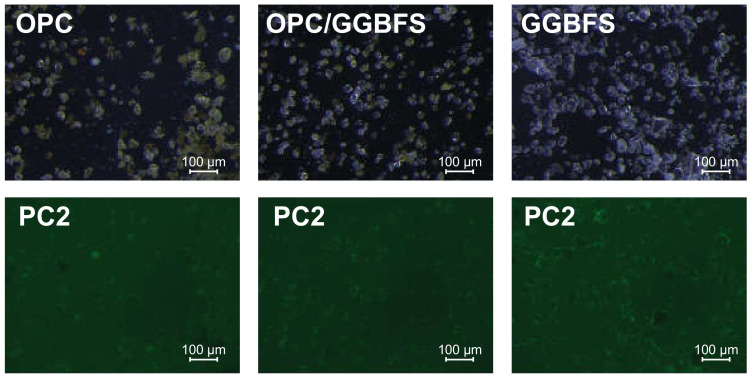
Light (**top**) and fluorescence (**bottom**) microscopic images of suspensions of OPC, OPC/GGBFS and GGBFS in synthetic pore solution with 1.2% bwob PC2.

Samples of coarse OPC, coarse GGBFS and a 50:50-mix of both were used with the four SPs in a concentration of 1.2% bwob for the fluorescence microscopic investigations. The *d*_50_ of the particles is about 40 µm. Representatively, Figure 8 shows the light and fluorescence microscopic images of the three substrates.

**Figure 9:**
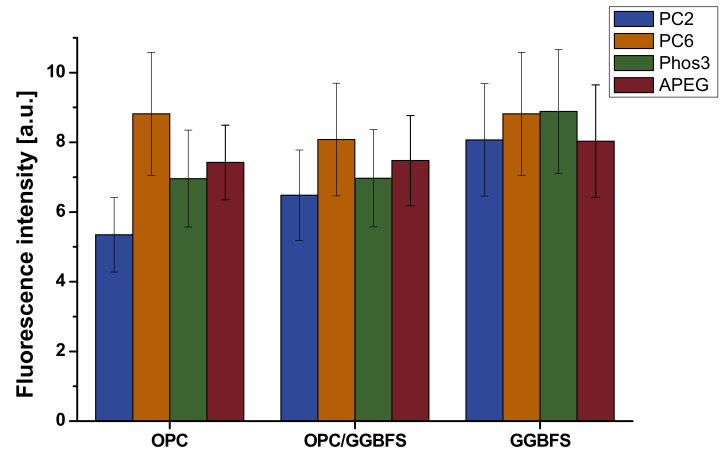
Analyzed fluorescence intensity at particles depending on material and SP (1.2% bwob).

The light microscopic images indicate that an optical separation of cement and slag particles is quite challenging. Slag particles are widely transparent and of very angular shape while the cement particles are mixed of different colored or transparent and different shaped ones. It is not promising to carry out selective analysis of the fluorescence of the mixed sample, so all images were analyzed in the same way: After subtracting the background signal, representing not adsorbed SP-polymer, the residual fluorescence of the particles were analyzed by imageJ. The mean intensity of the fluorescence signal of every pixel is taken as basis for further discussion. The results are illustrated in Figure 9. It is obvious that sample preparation and image selection have a great impact on the outcome of the analysis. Moreover, it can be seen that the fluorescence and thus SP-adsorption is quite inhomogeneous for most particles. The roughness of surface areas is obviously another issue that impacts the fluorescence signal. Overall, the error for these experimental data is estimated to 20% considering standard deviation of multiple image analysis, thus reliable quantitative statements are hard to make. However, PC6 shows the most adsorption to OPC, while PC2 shows the less. For pure GGBFS, only negligible differences in SP-adsorption are recognizable; PC6 and Phos3 show a slightly higher signal than PC2 and APEG, but, in comparison to OPC, PC2 adsorbs much better. The blended suspension shows for PC6, Phos3 and APEG quite similar values as the OPC samples, but PC2 shows significantly more adsorption.

The quite similar adsorption to GGBFS probably occurs due to the homogeneity of the material. Although Habbaba et al. reported a higher affinity of SPs with high charge density to adsorb to GGBFS [25], the high ion concentration of the used synthetic pore solution diminishes any differences concerning SP–GGBFS interaction.

### 4.2. Slump Flow

**Figure 10:**
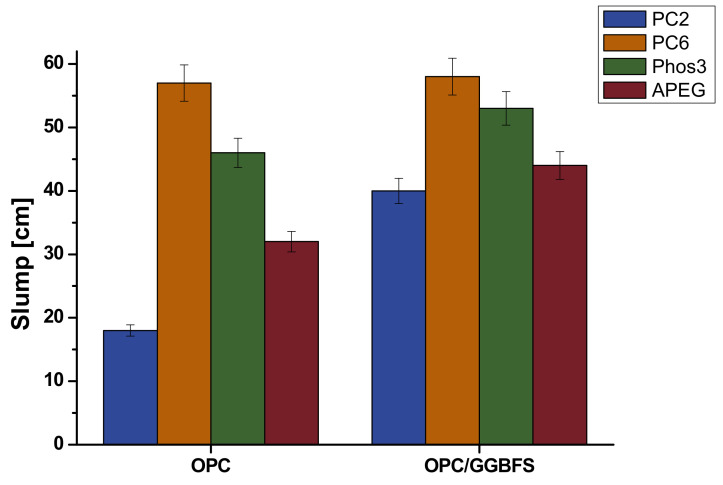
Slump of OPC and GGBFS-blended pastes with different SPs.

According to Table 4, pastes were mixed and the slump flow was measured. The results are given in Figure 10. The plasticizing effects of PC2 and PC6 have already been studied in ultra-high-performance concrete (Arend et al. [27]) and the results correspond well to the slump measurements with pure OPC. PC2 is not able to plasticize the paste to flowable consistency (18 cm), while PC6 generates a quite liquid paste (57 cm). Second best plasticizing effect shows Phos3 with 46 cm slump, followed by APEG with 32 cm. The blended paste shows overall higher slump values with the same order of SP-effectiveness. However, in the case of PC2, the plasticization increase is serious (40 cm). Phos3 and APEG show higher values as well but not as significant (53 and 44 cm). Within the error range (±5%), PC6 shows the same performance as for pure OPC (58 cm).

### 4.3. Correlation of Fluorescence Microscopy and Rheology

Considering the results of both experiments, a general correlation of microscopic detected SP-adsorption and plasticizing effect is possible. Especially for PC2 the least amount of detected adsorption correlates with the smallest slump flow of paste, while PC6 shows the highest values in both approaches. Increasing amounts of GGBFS generate the greatest increase for PC2—in terms of slump and SP-adsorption. Overall, the amount of polymer adsorbed to GGBFS is quite similar (Figure 9).

The grain size distribution and specific surface area of OPC and GGBFS is not taken into account in this experiment. Thus, only the coarse grain fraction is used for microscopic experiments. For the slump, the original GGBFS is slightly coarser than the cement resulting in a lower specific surface area and water demand, which may lead to higher slump values in general. The unproportioned high increase in the case of PC2 must be based on better adsorption and, thus, increase in slump flow. The method might be improved by reducing the differences between suspension and paste to reduce the number of interfering parameters. Further, the dosage of the SPs is very high due to the relation to UHPC, but lower dosages will be investigated for example to reduce the signal-noise-ratio concerning non-adsorbed polymer and reduced specific surface area.

## 5. Confocal Laser Scanning Microscopy (CLSM)

**Figure 11:**
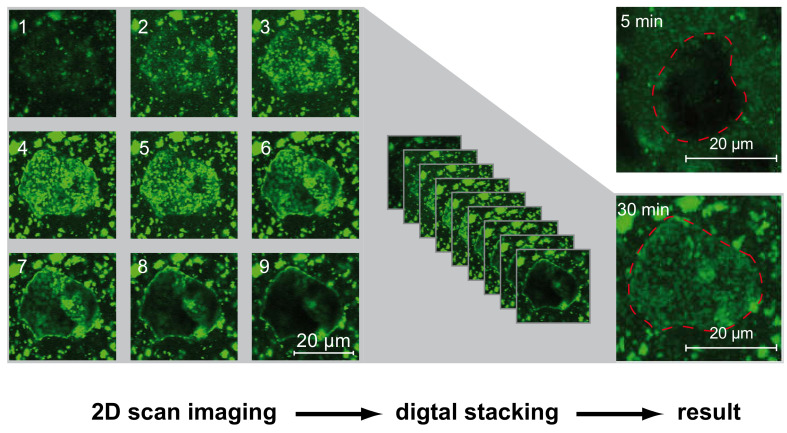
Stackable fluorescence images by CLSM of cement grains in Phos3-solution 5 and 30 min after mixing. On the left, the single plane images, which are stacked to the final images on the right. The red lines mark the grains.

As mentioned, the resolution of the fluorescence microscope used for the presented experiments is not sufficient for the detection of hydrate phases themselves. By using confocal laser scanning microscopy, the magnification can be increased by a factor of 10 and higher. Furthermore, the resolution and sharpness of images can be improved tremendously by confocal 3D imaging. Figure 11 shows a sketch of the CLSM imaging. As a result, two cement grains are shown, with one captured as quickly as possible after mixing (approximately 5 min due to preparation and device adjustment) and the other after 30 min in a basic solution of stained Phos3. Areas of bright fluorescence represent SP-adsorbing surfaces such as hydrate phases, which develop over time. Smaller particles are resolved as well.

As mentioned above, the interaction of SPs with early hydrate phases can be crucial for their performance. As reported for example by Pott et al. [41], the aluminum and silicon reaction of cement hydration can be affected by SPs in very different ways. Identifying particular clinker phases (C_3_A and C_3_S) prior to this laser microscopic investigation may lead to convincing in-situ results, especially if the possibility of using and imaging differently stained SP-types parallel is taken into account. Of course, the experimental effort is high but concerning the stagnancy of SP-understanding worth it.

## 6. Conclusions and Outlook

Although it is a dominating field of construction chemistry research, purposeful SP-development seems to stagnate recently. Many different phenomena and systems have been investigated, more or less satisfying explanations were found, but reliable transitions from one system to another are uncertain. Real progress is still widely based on empirical studies of specific materials, while many researchers try do develop efficient predicting models for all effects of SPs. In this context, fluorescence microscopy is a useful tool, whose development is at the beginning and further refinements are necessary to tap its whole potential. The unique ability to localize and quantify polymers in-situ enables a better link of scientific measurements to largescale applications. On this way, the first steps are to apply the new method to known phenomena and evaluate the outcome:

- The shown correlation present SP and retardation is in line with previous results obtained by other methods such as in-situ XRD. It was shown by fluorescence microscopic, calorimetric and electron microscopic investigations that the different chemical composition of the four SPs is responsible for the retardation of clinker hydration. The formation of passivating polymer layers is found by the results, but different extents of ion coordination in solution have to be considered as well to interpret all results.
- The amount of adsorbed polymer correlates in the conducted experiments roughly with rheological investigation, remembering this parameter is not standing alone in most cases. The best correlation was found for MPEG-type SPs (PC2 and PC6), while the APEG and phosphate-SP did not show sufficient differences for clear interpretation. It is assumed that the UHPC-specific high SP dosage is responsible and will be reduced in further work.
- The general approach to use CLSM in terms of high resolved in-situ experiments to investigate early hydration is presented as well, illustrating the potential of the approach.

Up to this point, several challenges need to be addressed in further work. Most of them have to be mentioned in terms of special sample preparation. Using the imaging method of fluorescence microscopy, many parameters have to be changed compared to real pastes to generate interpretable images. The comparability of the diluted suspensions of coarse binder particles to real pastes is not trivial. Grainsize distribution is a very important issue concerning specific surface area and reactivity, which cannot be concerned due to the required style of the fluorescence images, allowing digital analysis. Further, the ratio of used SP to the mass instead of the specific surface area of substrates is supposed to be a drastic simplification affecting the SP adsorption, because the amount of present SP tends to be too high. Using synthetic pore solution to compensate the high w/b values probably influences the processes of early hydration and should be investigated in terms of phase formation. It will be essential for the reliability of the fluorescence microscopic results to make sure that the conditions in investigated samples are as comparable as possible to the ones in corresponding pastes.

An even more general aspect is the staining of the polymers itself. Charge carrying groups are consumed to bind the fluorescent dye, thus the extent should be limited as much as possible to preserve the adsorption characteristics. On the other hand, strong fluorescence is required to allow a high sensitivity of the measurements, especially if low SP to substrate ratios are investigated. To find the best compromise the adsorption behavior of differently intensive stained and unstained polymers could be investigated in terms of adsorption isotherms via TOC.

Although some approaches reduce the complexity of ongoing processes concerning hydration and influence of SPs, such as delayed addition of SPs to the mixture (because early hydrates are already formed when SP is added [29]), the investigation in complicated conditions, such as the “direct-addition-scenario”, should be investigated further to improve for example mix designs of UHPC. Wherefore, beside all challenges, in-situ methods such as the fluorescence microscopy are predestinated.

## Figures and Tables

**Table 1:** Oxide composition determined by XRF.

Oxide Content [wt%]	SiO_2_	Al_2_O_3_	Fe_2_O_3_	CaO	MgO	K_2_O	Na_2_O	P_2_O_5_	SO_3_	Others	LOI
Silica fume	95.6	0.1	0.1	0.5	0.3	0.7	0.2	0.1	0.1	0.9	1.4
OPC	21.5	3.7	4.3	64.3	0.9	0.4	0.3	0.2	2.4	0.3	1.7
GGBFS	35.8	11.7	0.4	43.8	5.8	0.4	0.2	–	0.3	0.8	0.8

**Table 2:** Phases composition of used cement estimated by XRD and Rietveld refinement.

Phase	C_3_S	C_2_S	C_3_A	C_4_AF	Gypsum and Hemihydrate
Content [%]	61.3	15.9	3.9	13.9	3.3

**Table 3:** Composition of calorimetry samples. Grey samples are considered for microscopic investigation.

SP	%bwob	SP Conc. [wt%]	SP Total Mass [g]	C_3_S [g]	Silica Fume [g]	Water [g]	Syn. PS [g]
–	–	–	–	1.5	–	–	0.60
PC2	0.2	38	0.008	1.5	–	–	0.37
PC2	0.5	38	0.020	1.5	–	–	0.36
PC2	1.2	38	0.048	1.5	–	–	0.35
PC6	0.5	40	0.019	1.5	–	–	0.36
PC6	1.2	40	0.045	1.5	–	–	0.35
Phos3	0.5	33	0.023	1.5	–	–	0.36
Phos3	1.2	33	0.055	1.5	–	–	0.34
APEG	0.5	35	0.021	1.5	–	–	0.36
APEG	1.2	35	0.051	1.5	–	–	0.34
–	–	–	–	1.5	–	0.60	–
PC2	0.5	38	0.020	1.5	–	0.36	–
PC2	1.2	38	0.048	1.5	–	0.35	–
PC6	0.5	40	0.019	1.5	–	0.36	–
PC6	1.2	40	0.045	1.5	–	0.35	–
Phos3	0.5	33	0.023	1.5	–	0.36	–
Phos3	1.2	33	0.055	1.5	–	0.34	–
APEG	0.5	35	0.021	1.5	–	0.36	–
APEG	1.2	35	0.051	1.5	–	0.34	–
–	–	–	–	1.275	0.225	–	0.60
PC2	0.5	38	0.020	1.275	0.225	–	0.36
PC2	1.2	38	0.048	1.275	0.225	–	0.35
PC6	0.5	40	0.019	1.275	0.225	–	0.36
PC6	1.2	40	0.045	1.275	0.225	–	0.35
Phos3	0.5	33	0.023	1.275	0.225	–	0.36
Phos3	1.2	33	0.055	1.275	0.225	–	0.34
APEG	0.5	35	0.021	1.275	0.225	–	0.36
APEG	1.2	35	0.051	1.275	0.225	–	0.34
–	–	–	–	1.275	0.225	0.60	–
PC2	0.5	38	0.020	1.275	0.225	0.36	–
PC2	1.2	38	0.048	1.275	0.225	0.35	–
PC6	0.5	40	0.019	1.275	0.225	0.36	–
PC6	1.2	40	0.045	1.275	0.225	0.35	–
Phos3	0.5	33	0.023	1.275	0.225	0.36	–
Phos3	1.2	33	0.055	1.275	0.225	0.34	–
APEG	0.5	35	0.021	1.275	0.225	0.36	–
APEG	1.2	35	0.051	1.275	0.225	0.34	–

**Table 4:** Composition of pastes for slump.

SP	%bwob	SP Conc. [wt%]	SP Total Mass [g]	OPC [g]	GGBFS [g]	Water [g]
PC2	1.2	38	22.2	700	–	161.2
PC6	1.2	40	21.2	700	–	162.2
Phos3	1.2	33	25.5	700	–	158.0
APEG	1.2	35	24.0	700	–	159.4
PC2	1.2	38	22.2	350	350	161.2
PC6	1.2	40	21.2	350	350	162.2
Phos3	1.2	33	25.5	350	350	158.0
APEG	1.2	35	24.0	350	350	159.4

## Supplementary Materials

The following are available online at www.mdpi.com/xxx/s1, Figure S1: Diffractogramm of used C3S (black) with pattern (red) of C3S-reference PDF 31-301, Table S1: Composition of synthetic pore solution [51], Table S2: Data of Figure 4.
