# Supplementary material for: Fluorescence Microscopy of Superplasticizers in Cementitious Systems: Applications and Challenges
Source: Materials (Basel). 2020 Aug 24;13(17):3733. doi: 10.3390/ma13173733 (PMC7504373; doi:10.3390/ma13173733)
Supplement: Supplementary file 1 [file materials-13-03733-s001.pdf]

materials

Supplementary Materials: Fluorescence Microscopy of Superplasticizers in Cementitious Systems: Applications and Challenges

Johannes Arend *, Alexander Wetzel and Bernhard Middendorf

Department of structural materials and construction chemistry, University of Kassel, Mönchebergstr. 7, 34125 Kassel, Germany; alexander.wetzel@uni-kassel.de (A.W.); middendorf@uni-kassel.de (B.M.)

* Correspondence: j.arend@uni-kassel.de; Tel.: +49-561-804-2601

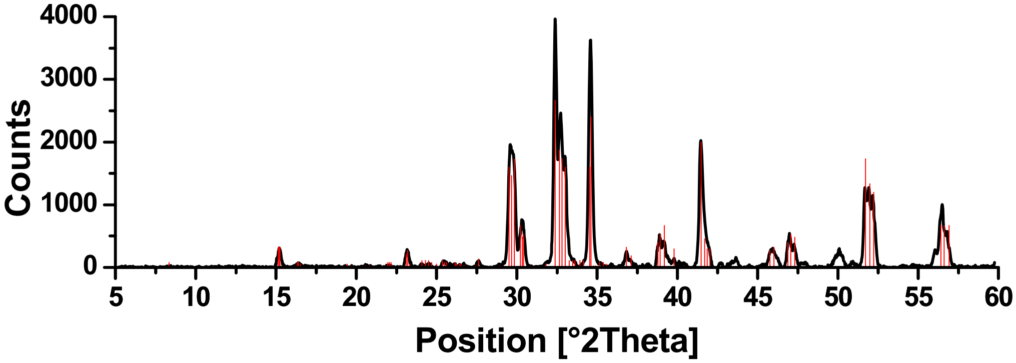

Figure S1. Diffractogramm of used C3S (black) with pattern (red) of C3S-reference PDF 31-301.

Table 1. Composition of synthetic pore solution [51].

Salt	[g/L]
$CaSO_4 \cdot 2\,H_2O$	1.72
Na_2SO_4	6.96
K_2SO_4	4.75
KOH	7.12

Table 2. Data of Figure 4.

Hydration peak in [h]	H_2O	Syn. PS	H_2O + SF	Syn. PS + SF
C_3S pure	12.5	9.0	11.0	7.5
PC2 0.5%	50.0	19.0	19.5	11.0
PC2 1.2%	93.5	24.0	44.5	15.0
PC6 0.5%	101.5	28.0	29.5	13.5
PC6 1.2%	>168.0	53.0	>168.0	26.0
Phos3 0.5%	33.5	23.5	17.5	11.0
Phos3 1.2%	81.5	48.5	35.5	20.5
APEG 0.5%	>168.0	>168.0	>168.0	53.5
APEG 1.2%	>168.0	>168.0	>168.0	>168.0

© 2020 by the authors. Licensee MDPI, Basel, Switzerland. This article is an open access article distributed under the terms and conditions of the Creative Commons Attribution (CC BY) license (http://creativecommons.org/licenses/by/4.0/).
